# Type 2 diabetes mellitus is associated with asymptomatic acute abdomen among elderly patients admitted to acute tertiary care hospital wards

**DOI:** 10.1007/s00592-026-02671-y

**Published:** 2026-03-17

**Authors:** Andrea Tumminia, Raffaella Romano, Francesco Frasca, Francesco Galeano, Roberto Baratta, Vittorio Oteri, Alessia Longo, Lucia Frittitta, Rosario Le Moli, Tommaso Piticchio, Antonino Di Pino, Maurizio Di Marco, Luigi Piazza, Maria Carolina Picardo, Paola Magnano San Lio, Filippo Luca Fimognari, Marcello Romano

**Affiliations:** 1 Endocrine Unit, Garibaldi-Nesima Hospital, Catania, Italy; 2 Geriatrics Unit, Garibaldi-Nesima Hospital, Catania, Italy; 3 Department of Clinical and Experimental Medicine, Endocrinology Section, Garibaldi-Nesima Hospital, University of Catania, Catania, Italy; 4 Diabetes and Obesity Center, Garibaldi-Nesima Hospital, University of Catania, Catania, Italy; 5 Department of Medicine and Surgery, University Kore of Enna, Enna, Italy; 6 Unit of Diabetology, Metabolic and Endocrine Diseases, “Cannizzaro” Emergency Hospital, Catania, Italy; 7 Department of Clinical and Experimental Medicine, Internal Medicine, Garibaldi-Nesima Hospital, University of Catania, Catania, Italy; 8 Department of General and Emergency Surgery, Garibaldi-Nesima Hospital, Catania, Italy; 9 Department of General Surgery, Cannizzaro Hospital, Catania, Italy; 10 Department of Clinical and Experimental Medicine, University of Catania, Catania, Italy; 11 Unit of Geriatrics, Department of Medicine, Azienda Ospedaliera “Annunziata - Mariano Santo - S. Barbara”, Cosenza, Italy

**Keywords:** Type 2 diabetes, Acute abdomen, Elderly, Pain perception, Emergency medicine, Predictors

## Abstract

**Background:**

Pain may be absent in a substantial proportion of elderly patients with acute abdominal conditions. This study explored the association between type 2 diabetes mellitus (T2DM) and asymptomatic presentation.

**Methods:**

We conducted a cross-sectional analysis of 215 patients aged ≥ 65 years admitted with acute abdominal conditions. Demographic, clinical, and laboratory data were extracted from medical records. Descriptive statistics and multivariable logistic regression were used to identify associative predictors of asymptomatic acute abdomen (AAA).

**Results:**

The median age was 82 years [77–86]; 54.4% (*n* = 117) were female; 31.2% (*n* = 67) had T2DM. Overall, 33.5% (*n* = 72) presented without abdominal pain. T2DM prevalence was higher in AAA than symptomatic patients (44.4% vs. 24.5%, *p* < 0.01). In multivariable analysis, T2DM (OR 1.95, 95% CI 1.10–3.45, *p* = 0.02), lower heart rate (OR 0.83, 95% CI 0.71–0.96, *p* = 0.01), and absence of fever (OR 0.50, 95% CI 0.26–0.95, *p* = 0.03) were associated with AAA. Among patients with T2DM, longer diabetes duration (12.5 years [10.5–14.5] vs. 8.8 years [5.0–11.0]; *p* < 0.01) and higher HbA1c (8.2% [7.2–8.7] vs. 7.5% [6.8–7.6]; *p* = 0.02) were associated with asymptomatic presentation.

**Conclusions:**

Asymptomatic acute abdomen is common among elderly patients. Long-standing and poorly controlled T2DM is associated with absent pain. Prospective studies are needed to clarify causal mechanisms, and early glyco-metabolic assessment may aid recognition of at-risk patients.

## Introduction

Acute abdominal conditions represent one of the most frequent causes of hospital admission to emergency medical and surgical wards and continue to pose a significant clinical challenge worldwide [1, 2]. They encompass a broad spectrum of pathologies, ranging from inflammatory to ischemic and obstructive processes, many of which require urgent diagnosis and timely intervention [3]. Their incidence rises steadily with age, and in older adults these conditions are often associated with a more complex clinical course, greater healthcare resource utilization, and increased postoperative morbidity and mortality [4, 5].

A critical issue in this population is the atypical clinical presentation [6]. In elderly patients, classical symptoms are often blunted or absent, which may delay recognition of the underlying disease. Instead of abdominal pain, which is traditionally regarded as the cardinal feature, older adults may present with nonspecific signs such as confusion, anorexia, nausea, vomiting, or even falls [6, 7]. Notably, up to one-third of elderly patients with acute abdomen have been reported to present without abdominal pain, particularly among frail and multimorbid individuals [8, 9]. This condition, hereafter defined as asymptomatic acute abdomen (AAA), is strongly associated with diagnostic delays, inappropriate treatment, and higher mortality [10].

Several factors have been proposed to contribute to asymptomatic acute abdomen (AAA) in older adults, including age-related changes in nociception, reduced visceral sensitivity, and the cumulative burden of comorbidities such as diabetes mellitus [11]. Type 2 diabetes mellitus (T2DM), which is highly prevalent in the elderly population, is associated with chronic microvascular and neuro-metabolic complications, including peripheral neuropathy and autonomic dysfunction [12–14]. These conditions have been linked to altered pain perception and modified autonomic responses, which may contribute to atypical or attenuated clinical presentations of acute abdominal conditions [15–18]. Conceptually, this phenomenon has been compared to silent myocardial ischemia in patients with diabetes [16]. This analogy, which is intended to support biological plausibility rather than to suggest identical pathophysiological mechanisms, have been described in gastrointestinal emergencies as well [17, 18].

Despite these observations, few studies have systematically evaluated the relationship between T2DM, glycemic control, and AAA across different acute abdominal conditions [19, 20]. In particular, the influence of diabetes duration and poor glycemic control on the likelihood of atypical presentations remains largely unexplored [21].

The aim of this cross-sectional analysis was to determine the prevalence of AAA in a population of elderly patients admitted with acute abdominal conditions and to identify predictors of asymptomatic presentation. We hypothesized that T2DM, especially when long-standing and poorly controlled, would be associated with an increased probability of AAA.

## Methods

This was a cross-sectional study including all the patients aged ≥ 65 years that were discharged, between January 2022 and December 2023, with a diagnosis of acute abdomen, from two tertiary care hospital wards (the Geriatrics Unit and the General and Emergency Surgery Unit of the Garibaldi-Nesima Hospital, Catania, Italy).

Inclusion criteria were: (a) age ≥ 65 years; (b) hospital admission due to an acute abdominal condition confirmed by imaging, endoscopy, or surgery. Exclusion criteria were: (a) incomplete clinical records; (b) non-abdominal acute conditions misclassified at triage.

Demographic characteristics, comorbidities, chronic medications, vital signs at admission, laboratory data, imaging findings, and final diagnosis were extracted from the patients’ medical records. The presence of abdominal pain at presentation was determined from triage documentation and coded dichotomously as present or absent (e.g. AAA).

The following baseline clinical, anthropometric and biochemical variables were evaluated: body weight, height, body mass index (BMI), systolic blood pressure (SBP), diastolic blood pressure (DBP), fasting plasma glucose (FPG), glycated hemoglobin (HbA1c, determined by high-performance liquid chromatography), total cholesterol, high density lipoprotein cholesterol (HDL-C), low density lipoprotein cholesterol (LDL-C, calculated with Friedewald formula if triglycerides value was lower than 400 mg/dl) [22], triglycerides (TG), creatinine levels, estimated glomerular filtration rate (according to the Chronic Kidney Disease Epidemiology Collaboration formula) [23], albumin-to-creatinine ratio (ACR), glutamic oxaloacetic transaminase (GOT), glutamic pyruvic transaminase (GPT), amylase, lipase, C-reactive protein (CRP), procalcitonin (PCT).

The presence of different comorbidities: hypertension (SBP ≥ 140 mmHg and/or DBP ≥ 90 mmHg, or taking antihypertensive medication), established cardiovascular disease (e.g. myocardial infarction, heart failure, stroke), chronic kidney disease (e.g. if either eGFR < 60 ml/min or ACR > 30 mg/g), T2DM, obesity (BMI ≥ 30 kg/m^2^), dyslipidemia (defined according to the 2025 focused update of the 2019 ESC/EAS guidelines [24] or by already taking lipid-lowering drugs at the time of hospital admission), malignancies and chronic obstructive pulmonary diseases (COPD). Patients’ smoking habit and concomitant medications were also recorded.

The main study outcome was the evaluation of the absence/presence of abdominal pain at presentation according to different comorbidities.

Diabetes duration was calculated from the year of diagnosis. Secondary analyses were restricted to the diabetic subgroup to evaluate associations between AAA, diabetes duration, and HbA1c levels at hospital ward admission.

The study was conducted in accordance with the principles of the Declaration of Helsinki and its later amendments [25]. Ethical committee approval was not required according to institutional regulations for retrospective analyses of anonymized data. For this type of study, informed consent was not required.

### Statistical analysis

The available population determined the sample size; analyses should be therefore regarded as exploratory and hypothesis-generating. Given the clinical context and the risk of skewed distributions in older acute-care patients, continuous variables were primarily summarized as median and interquartile range [IQR]. Group comparisons were performed using non-parametric methods. Sensitivity analyses using parametric methods were conducted where appropriate to confirm robustness. Categorical variables are presented as absolute numbers and percentages.

Comparisons between groups (e.g. AAA vs. pain at presentation) were performed using the Mann–Whitney U test for continuous variables, and the chi-square or Fisher’s exact test for categorical variables, as appropriate.

Univariate analyses were conducted for descriptive purposes only. Multivariable logistic regression analysis was used to explore factors associated with AAA and represents the primary analysis. The adjustment set was pre-specified a priori based on clinical relevance and data availability, and included age, sex, T2DM, heart rate, and presence of fever. All covariates were entered simultaneously into the model; no data-driven variable selection procedures were applied. Model performance metrics were not used for prediction purposes. Continuous predictors were modeled linearly due to sample size constraints. Analyses were conducted using a complete-case approach.

A two-sided p-value < 0.05 was considered statistically significant. Statistical analyses were carried out using STATA software, version 18.0 (StataCorp, College Station, TX, USA).

## Results

A total of 215 patients aged ≥ 65 years were analyzed, with a median age of 82 years [77–86]; 54.4% (*n* = 117) were female and 31.2% (*n* = 67) were affected by T2DM. Overall, 72 patients (33.5%) presented without abdominal pain. The prevalence of T2DM was significantly higher in patients with AAA compared to those experiencing abdominal pain (44.4% vs. 24.5%, *p* < 0.01). Clinical, anthropometrical and biochemical characteristics of the study population according to pain perception are summarized in Table 1. Patients with AAA displayed higher FPG (135 mg/dl [121–154] vs. 104 mg/dl [92–121]; *p* = 0.02), higher HbA1c levels (8.2% [7.2–8.7] vs. 7.5% [6.8–7.6]; *p* = 0.02), and a longer duration of diabetes (12.5 years [10.5–14.5] vs. 8.8 [5.0–11.0]; p < < 0.01) compared to those experiencing abdominal pain.

**Table 1:** Clinical and biochemical variables of the study population and according to pain perception

Patients’ characteristics	Overall (*n* = 215)	Asymptomatic (*n* = 72)	Symptomatic (*n* = 143)	*p*
*Demographic and anthropometrical*				
Age, years (median [IQR])	82 [77–86]	84 [78–88]	81 [76–85]	0.21
Female sex, n (%)	117 (54.4)	40 (55.6)	77 (53.8)	0.81
BMI, Kg/m^2,^ (median [IQR])	25.8 [23.1–28.9]	25.1 [22.9–28.6]	26.2 [23.3–29.1]	0.29
Smokers, n (%)	38 (17.7)	11 (15.3)	27 (18.9)	0.52
*Comorbidities*				
T2DM, n (%)	67 (31.2)	32 (44.4)	35 (24.5)	< 0.01
Diabetes duration, years (median [IQR])*	10.0 [7.0–13.0]	12.5 [10.5–14.5]	8.8 [5.0–11.0]	< 0.01
Obesity, n (%)	40 (18.6)	13 (18.1)	27 (18.9)	0.92
Dyslipidemia, n (%)	92 (42.8)	28 (38.9)	64 (44.8)	0.42
Hypertension, n (%)	141 (65.6)	48 (66.7)	93 (65.0)	0.81
Atrial fibrillation, n (%)	46 (21.4)	17 (23.6)	29 (20.3)	0.57
COPD, n (%)	18 (8.4)	6 (8.3)	12 (8.4)	0.95
Malignancies, n (%)	11 (5.1)	4 (5.6)	7 (4.9)	0.78
Cardiovascular diseases, n (%)**	58 (27.0)	20 (27.8)	38 (26.6)	0.86
Chronic kidney disease, n (%)	38 (17.7)	15 (20.8)	23 (16.1)	0.39
*Clinical and biochemical variables*				
FPG, mg/dL (median [IQR])	118 [102–138]	135 [121–154]	104 [92–121]	0.02
HbA1c, % (median [IQR])*	7.6% [7.0–8.2]	8.2% [7.2–8.7]	7.5% [6.8–7.6]	0.02
SBP, mmHg (median [IQR])	132 [120–144]	131 [120–141]	133 [120–145]	0.55
DBP, mmHg (median [IQR])	74 [67–81]	74 [66–80]	75 [68–82]	0.62
Total cholesterol, mg/dL (median [IQR])	181 [155–207]	179 [150–203]	182 [156–210]	0.60
HDL-C, mg/dL (median [IQR])	47 [37–55]	45 [39–56]	49 [36–55]	0.41
LDL-C, mg/dL (median [IQR])	78 [59–97]	76 [58–94]	79 [59–99]	0.58
Triglycerides, mg/dL (median [IQR])	142 [110–189]	138 [108–182]	145 [112–191]	0.48
Creatinine, mg/dL (median [IQR])	1.18 [0.80–1.48]	1.15 [0.81–1.43]	1.19 [0.79–1.51]	0.56
GOT (AST), U/L (median [IQR])	28 [19–36]	25 [19–35]	30 [19–36]	0.34
GPT (ALT), U/L (median [IQR])	30 [19–39]	29 [20–38]	31 [19–41]	0.51
Amylase, U.I./L (median [IQR])	55 [40–78]	53 [39–75]	57 [41–80]	0.47
Lipase, U.I./L (median [IQR])	48 [30–70]	46 [28–68]	49 [32–71]	0.53
CRP, mg/L (median [IQR])	32 [18–61]	30 [17–58]	34 [19–64]	0.39
PCT, ng/mL (median [IQR])	0.32 [0.12–0.85]	0.30 [0.10–0.80]	0.34 [0.14–0.90]	0.44
Heart rate, bpm (median [IQR])	85 [74–97]	80 [69–92]	88 [77–99]	0.04
Fever ≥ 38 °C, n (%)	89 (41.4)	22 (30.6)	67 (46.9)	0.02
White blood cells, ×10⁹/L (median [IQR])	10.8 [5.4–12.9]	10.2 [5.3–13.9]	11.1 [5.6–13.0]	0.11
Hemoglobin, g/dL (median [IQR])	11.9 [9.0–12.9]	11.7 [9.1–13.1]	12.1 [8.8–13.0]	0.29
Platelets, ×10⁹/L (median [IQR])	240 [150–265]	231 [155–260]	248 [148–261]	0.43
*Concomitant medications*				
ARBs, n (%)	60 (27.9)	19 (26.4)	41 (28.7)	0.72
ACE‑I, n (%)	45 (20.9)	13 (18.1)	32 (22.4)	0.46
Beta blockers, n (%)	50 (23.3)	15 (20.8)	35 (24.5)	0.55
CCB, n (%)	35 (16.3)	10 (13.9)	25 (17.5)	0.45
Aspirin, n (%)	70 (32.6)	22 (30.6)	48 (33.6)	0.66
Statins, n (%)	80 (37.2)	25 (34.7)	55 (38.5)	0.57
Ezetimibe, n (%)	8 (3.7)	3 (4.2)	5 (3.5)	0.78
Fibrates, n (%)	5 (2.3)	1 (1.4)	4 (2.8)	0.67
Insulin therapy, n (%)*	20 (29.9)	10 (31.3)	10 (28.6)	0.78
Metformin, n (%)*	30 (44.8)	13 (40.6)	17 (48.6)	0.43

Data are presented as median and interquartile range [IQR] for continuous variables or numbers and percentages (%) for categorical variables

*FPG* fasting plasma glucose, *HbA1c* glycated hemoglobin, *SBP* systolic blood pressure, *DBP* diastolic blood pressure, *HDL-C* HDL cholesterol, *LDL-C* LDL cholesterol, *COPD* chronic obstructive pulmonary disease; ECD, *GOT* glutamic oxaloacetic transaminase, *GPT* glutamic pyruvic transaminase, *CRP* C-reactive protein, *PCT* procalcitonin, *ARBs* angiotensin II receptor blockers, *ACE-I* angiotensin-converting enzyme inhibitors, *CCB* calcium channel blockers

*Available for diabetic patients only. **Cardiovascular diseases comprise myocardial infarction, heart failure and stroke

The most frequent acute abdominal conditions were cholecystitis (18.6%), diverticulitis (14.4%), gastrointestinal hemorrhage (13.0%), intestinal obstruction (7.4%), acute appendicitis (5.6%), bowel perforation (4.2%), acute diverticulitis (3.7%), acute pancreatitis (3.7%) and kidney stones (3.3%), with no differences in their prevalence between the asymptomatic and symptomatic group (Table 2).

**Table 2:** Prevalence of different acute abdominal conditions in our population

Diagnosis	Overall (*n* = 215)	Asymptomatic (*n* = 72)	Symptomatic (*n* = 143)	*p*-value
Cholecystitis (n, %)	40 (18.6)	16 (22.2)	24 (16.8)	0.33
Diverticulitis (n, %)	31 (14.4)	10 (13.9)	21 (14.7)	0.87
Gastrointestinal hemorrhage (n, %)	28 (13.0)	10 (13.9)	18 (12.6)	0.79
Intestinal obstruction (n, %)	16 (7.4)	5 (6.9)	11 (7.7)	0.84
Appendicitis (n, %)	12 (5.6)	2 (2.8)	10 (7.0)	0.20
Bowel perforation (n, %)	9 (4.2)	4 (5.6)	5 (3.5)	0.48
Acute diverticulitis (n, %)	8 (3.7)	2 (2.8)	6 (4.2)	0.60
Acute pancreatitis (n, %)	8 (3.7)	3 (4.2)	5 (3.5)	0.80
Kidney stones (n, %)	7 (3.3)	2 (2.8)	5 (3.5)	0.78
Other conditions (n, %)	56 (26.0)	18 (25.0)	38 (26.6)	0.80

Data are presented as numbers and percentages (%)

On multivariate logistic regression analysis several variables were associated with AAA, including T2DM (OR 1.95, 95%CI 1.10–3.45, *p* = 0.02), lower heart rate (OR 0.83, 95%CI 0.71–0.96, *p* = 0.01) and the absence of fever (OR 0.50, 95%CI 0.26–0.95, *p* = 0.03) (Table 3).

**Table 3:** Multivariate logistic regression analysis for predictors of asymptomatic acute abdomen (AAA)

Variable	Odds ratio	95% CI	*p*-value
Type 2 diabetes mellitus	1.95	1.10–3.45	0.02
Heart rate	0.83	0.71–0.96	0.01
Fever	0.50	0.26–0.95	0.03
Female sex	0.95	0.56–1.60	0.84
Age	1.01	0.97–1.05	0.58

Odds ratios and 95% confidence intervals (CIs) are derived from a multivariable logistic regression model including age, sex, type 2 diabetes mellitus, heart rate, and fever. The model was pre-specified and exploratory in nature

**Fig. 1:**
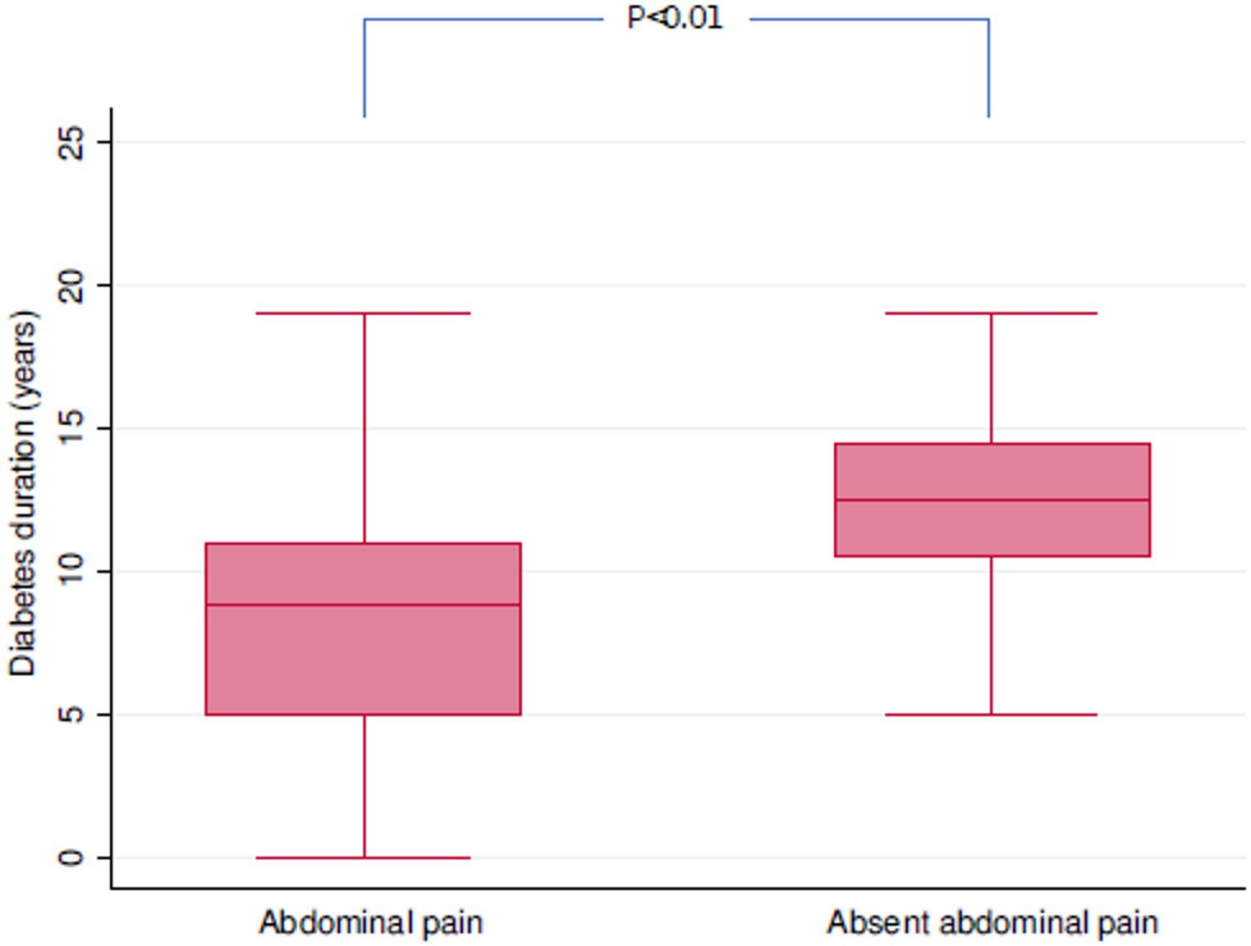
Distribution of diabetes duration according to abdominal pain presentation. Boxplots represent unadjusted distributions of diabetes duration in elderly patients with type 2 diabetes mellitus presenting with asymptomatic acute abdomen or with abdominal pain. Medians and interquartile ranges are displayed

**Fig. 2:**
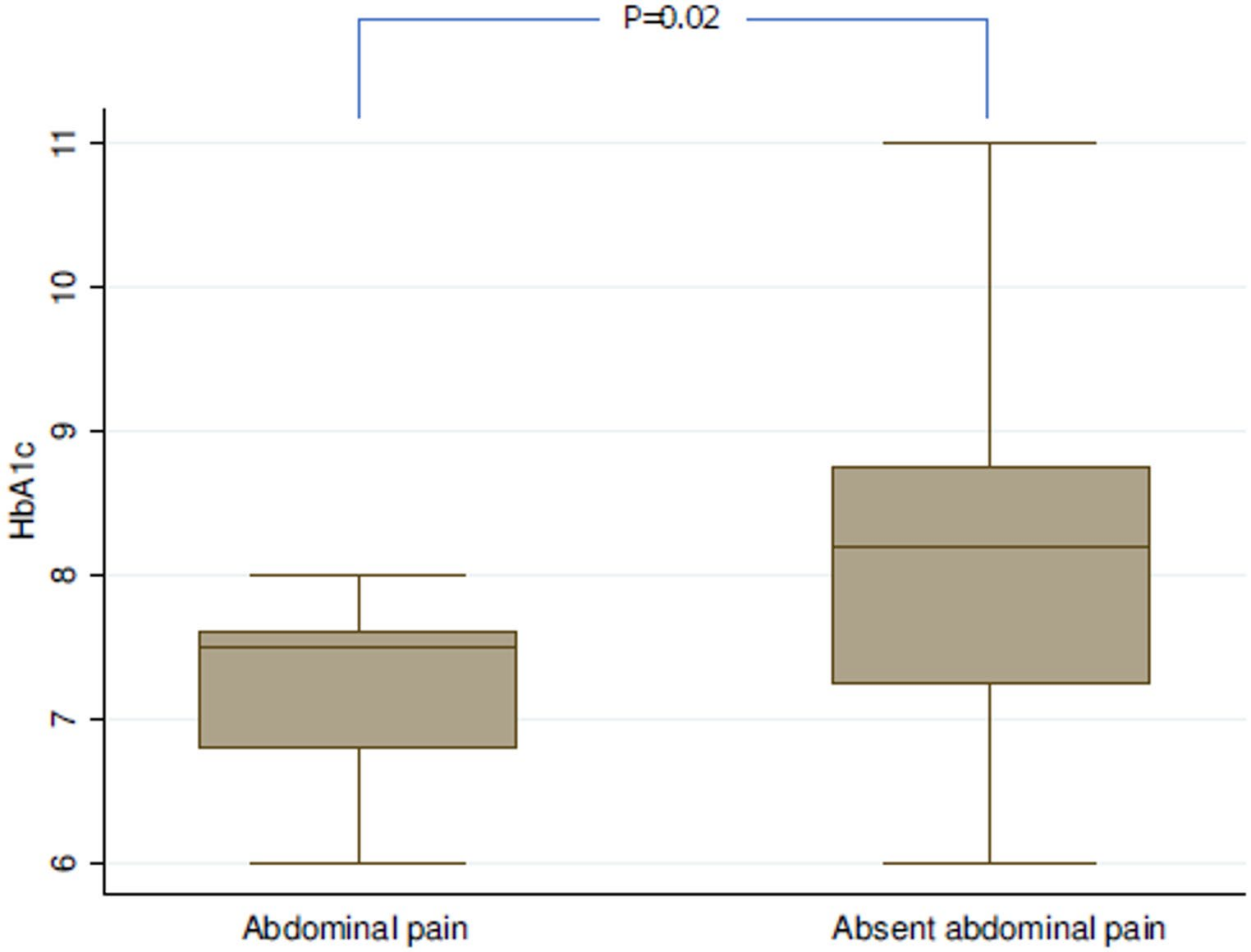
Distribution of HbA1c levels at ward admission according to abdominal pain presentation. Boxplots represent unadjusted distributions of glycated hemoglobin (HbA1c, %) in diabetic patients presenting with asymptomatic acute abdomen or abdominal pain. Medians and interquartile ranges are displayed

Further evaluations were limited to the subgroup of patients with T2DM (*n* = 67). Figures 1 and 2 show the boxplot distribution of diabetes duration and HbA1c levels at ward admission according to abdominal pain presentation. Specifically, patients presenting with asymptomatic acute abdomen exhibited a longer duration of diabetes compared with those reporting abdominal pain (12.5 years [10.5–14.5] vs. 8.8 years [5.0–11.0], *p* < 0.01). Similarly, HbA1c levels at ward admission were higher among asymptomatic patients than among those with symptomatic presentation (8.2% [7.2–8.7] vs. 7.5% [6.8–7.6], *p* = 0.02). These boxplots illustrate unadjusted distributions and highlight variability and overlap between groups; therefore, they should be interpreted as descriptive representations rather than evidence of dose–response or causal relationships.

## Discussion

In this cross-sectional analysis, approximately one-third of elderly patients with acute abdominal conditions presented without abdominal pain. This prevalence aligns with previous reports describing atypical presentations in 20–35% of older adults [8–10, 26].

The key finding of our study is the association between T2DM and pain absence. Nearly half of diabetic patients were asymptomatic, compared with only one quarter of non-diabetic individuals. This observation supports previous evidence of altered nociception in diabetes [12–14]. However, given the retrospective observational design, these findings should be interpreted as associative rather than causal. The study identifies clinical variables associated with asymptomatic presentation but does not allow inference on underlying mechanisms.

From a pathophysiological perspective, long-standing and poorly controlled diabetes has been associated with altered visceral pain perception through metabolic and neurovascular mechanisms, including chronic hyperglycemia-related nerve damage and autonomic dysfunction [27–30]. Although our study did not directly assess neuropathy, diabetes duration and HbA1c represent clinically meaningful proxies of cumulative metabolic burden. In this context, the association between higher HbA1c levels, longer disease duration, and absent abdominal pain observed in our subgroup analysis supports the biological plausibility of blunted visceral nociception in diabetic patients, without allowing mechanistic inference [31–33].

Beyond peripheral diabetic neuropathy, altered pain perception in older adults has been described in association with changes in central pain processing and age‑related modifications in nociceptive integration. Recent studies in geriatric populations have highlighted age‑associated changes in pain thresholds and functional connectivity of pain modulatory networks, which may contribute to atypical or blunted symptom presentation in older individuals [34–36].

It is known that unfavorable metabolic conditions may lead to an increased mortality in patients admitted to acute care wards [37]. Clinically, the absence of abdominal pain should not provide reassurance in elderly patients with diabetes. A high index of suspicion is warranted, and diagnostic strategies should emphasize early imaging and laboratory evaluation in this high-risk group [20, 38, 39]. Moreover, careful consideration of pharmacologic profiles is warranted, since medications influencing volume status or autonomic tone, may further alter hemodynamic responses and symptom perception in elderly diabetic patients [40]. Incorporating patients’ glycemic control and diabetes history into the initial assessment might facilitate timely recognition of atypical cases and prevent diagnostic delay.

Our findings also have implications for surgical decision-making. Previous studies have reported poorer outcomes and higher mortality among elderly individuals undergoing emergency surgery for abdominal conditions [18, 41]. Frailty, multimorbidity, and atypical presentations contribute to these adverse outcomes [5, 42]. Heightened awareness of AAA, particularly in diabetic patients, may improve triage, expedite intervention, and ultimately enhance prognosis.

The observed relationship between lower heart rate and absent pain perception likely reflects underlying autonomic dysfunction. In elderly patients, especially those with long-standing diabetes, autonomic neuropathy can blunt sympathetic activation and attenuate the chronotropic response to nociceptive stimuli. Aging itself further diminishes baroreflex sensitivity and cardiovascular reactivity, thereby contributing to clinically silent presentations [41].

Similarly, the lack of fever as an independent predictor of AAA may be explained by immune-senescence and blunted inflammatory responses in older adult [4, 41]. Elderly patients often exhibit impaired cytokine release and attenuated febrile reactions to infection or inflammation. Moreover, chronic comorbidities such as diabetes and cardiovascular disease can further suppress systemic inflammatory activation, meaning that the absence of fever does not exclude serious intra-abdominal pathology in this population [1, 4].

It is important to underline that these associations should be interpreted at an associative level, acknowledging that peripheral neuropathy, autonomic dysfunction, and alterations in central pain processing and age-related nociceptive integration may all contribute to blunted visceral pain perception in older adults.

While the association between diabetes and atypical abdominal symptoms has been previously described, our study adds value by highlighting the potential roles of diabetes duration and glycaemic control. Other strengths of this study comprise the inclusion of multiple acute abdominal diagnoses, and the accurate metabolic characterization of the studied patients.

Nevertheless, several limitations should be acknowledged. An important limitation of this study concerns the definition of asymptomatic acute abdomen, which was based exclusively on triage documentation reporting the absence of abdominal pain. No standardized pain assessment scale was systematically applied, and information on cognitive status, delirium, dementia, or communication impairments was not available. In an elderly population, these factors may substantially affect pain reporting and introduce a risk of misclassification bias, potentially leading to underestimation or misclassification of symptomatic presentations. Consequently, this limitation restricts the internal validity of the study and should be considered when interpreting the observed associations. Furthermore, all participants were Caucasian, which restricts the applicability of our findings to ethnically diverse populations. Finally, several potentially relevant confounders could not be included in the multivariable analysis, including the use of beta-blockers or other chronotropic drugs, presence of diabetic neuropathy or autonomic dysfunction, frailty indices, and opioid or analgesic use prior to admission. The absence of these variables may have influenced the observed associations, particularly those involving heart rate and pain perception, and represents a limitation of the study.

In conclusion, asymptomatic acute abdomen represents a common clinical presentation among elderly patients admitted to hospital wards. Among elderly individuals with T2DM, asymptomatic presentation was more frequently observed in association with longer disease duration and higher HbA1c levels. These findings identify a clinically relevant association that may assist in recognizing patients at increased risk of delayed diagnosis. Given the retrospective observational design, causal relationships and underlying mechanisms cannot be inferred and should be explored in future prospective studies.
